# The effects of sleep disturbance on a songbird’s vocal performance

**DOI:** 10.1098/rspb.2025.1409

**Published:** 2025-08-06

**Authors:** Juliane Gaviraghi Mussoi, Rebecca A. MacQueen, Margaret C. Stanley, Simeng Li, Cleo T. Quann, Kristal E. Cain

**Affiliations:** ^1^ School of Biological Sciences, The University of Auckland, Waipapa Taumata Rau, Auckland 1010, New Zealand; ^2^ School of Science, University of Waikato, Te Whare Wānanga o Waikato, Hamilton 3216, New Zealand; ^3^ AuLin College, Northeast Forestry University, Harbin 150040, People's Republic of China

**Keywords:** sleep, disturbances, songbirds, vocal communication, song complexity, common mynahs

## Abstract

Vocal communication serves several critical functions, such as species recognition, mate attraction and resource defence. However, environmental and physiological factors like urbanization, noise pollution and stress can negatively affect vocal performance, especially in birds. While several studies have demonstrated the effects of various disturbances on bird vocalizations, very few have tested how lack of sleep affects birdsong. To investigate the importance of sleep for adult birds’ vocalizations, we analysed the vocal performance of captive common mynahs (*Acridotheres tristis*) after three sleep disturbance experiments: the entire night, first 6 h of the night or last 6 h of the night of sleep disturbance. Sleep disturbances altered song performance, specifically by reducing song output and complexity, but did not affect call output. Sleep disturbances also affected call spectral parameters. These effects varied according to the degree of disturbance, strongest in a full night of sleep disturbance, followed by the first 6 h of the night and last 6 h of the night, respectively. These results indicate that even short-term sleep disturbance can affect adult birds’ vocalizations’ frequency and structure. These changes could alter birds’ communication, negatively impacting social interactions and the acquisition of resources and mates, potentially affecting fitness.

## 1. Introduction

Birds’ vocal communication is an exceptionally diverse and complex behaviour. Birds use vocalizations to attract mates, to acquire and maintain resources, and for individual and species recognition [1,2]. Bird vocal production requires the coordination of multiple complex systems (e.g. neuronal, respiratory and muscular) and is subject to performance limits [3,4]. Vocal performance parameters (such as duration, output, trill rate, frequency modulation and repertoire) can be related to and affected by nutritional condition [5,6], dominance [7], body size [8], cognitive ability [9], longevity [10] and motivation [11]. Therefore, vocal performance is often considered an honest signal of individual quality, condition and motivational state. However, vocal production and performance can also be affected by intrinsic and extrinsic factors, such as light pollution [12], noise pollution [2,13], habitat density [14] and stress [15], particularly early in life [16]. At least some of these effects may be mediated by the effects of sleep disturbance (SD) on vocal performance [17,18].

Sleep is essential for the performance of many waking behaviours, but birds’ sleep patterns can be disrupted by many things, including light [19], noise [20], migration [21] and predators [22]. Lack of sleep can impair coordination, motivation, attention, cognitive performance and memory [18,23–26]. In humans, research has shown that lack of sleep has large effects on vocal communication. In response to sleep deprivation, speech becomes more monotonic and word generation and fluency deteriorate, impairing the ability to verbalize thoughts and concepts [27–29]. The level of speech impairment is dependent on the degree of sleep deprivation (hours of sleeplessness) [30]. Avian communication has very similar neural and genetic underpinnings to humans, and vocal learning in birds is a key model for understanding human speech development [31,32]. Consequently, we have recently argued that these lines of evidence suggest that SD will likely have consequences for avian communication and that birds may be key model species for understanding the relationships between sleep and vocal communication [17]. However, surprisingly, little is known about the effects of the lack of sleep on vocal communication in birds [17].

Sleep has been demonstrated to play a role in song learning in young zebra finches (*Taeniopygia guttata*) [33] and varied tits (*Poecile varia*) [34], as well as in auditory discrimination in adult European starlings (*Sturnus vulgaris*) [35,36]. To date, only one study has directly investigated the effects of SD on bird vocalization; compared with an undisturbed night of sleep, a group of experimentally sleep-deprived Australian magpies (*Gymnorhina tibicen*) sang fewer but longer songs and changed their daily singing pattern [18]. This study investigated the effects of 12 h of sleep deprivation on song output [18]; however, response to SD likely varies depending on the type of vocalization (song and calls) and the length of the disturbance, and between species, sex and even individuals [17].

Here, we investigated the effects of different levels of sleep loss on vocal performance in an open-ended vocal learner, the common mynah (*Acridotheres tristis*). We analysed song and contact call output, call spectral properties and song-complexity parameters before and after birds had their sleep disturbed for three time periods (the entire night, first 6 h of the night and last 6 h of the night). We also compared the birds’ activity and resting levels after each treatment. We predicted common mynahs would be less active and less motivated to vocalize after SD than after a regular night, leading to reduced song output and less complex songs. We also predicted that birds would call less often, but because calls are largely innate, we predicted that contact calls would exhibit little to no spectral differences among treatments. Finally, we predicted that 12 h of SD would have a larger effect on mynah vocal performance than 6 h; yet, disturbances in the first half of the night would be less detrimental as it allows time for sleep recovery. If sleep disruptions affect vocalizations, birds residing in areas with high levels of disturbance (e.g. light and noise pollution at night) may experience changes in communication patterns, potentially impacting their reproductive success and survival.

## 2. Material and methods

### (a) Study species and housing

Thirteen wild adult common mynahs (seven females and six males) were caught in the Auckland region of New Zealand in September 2020 (Austral spring) using handmade, PeeGee-style traps baited with dog food pellets [37]. Birds were transported and held inside a temperature-controlled (23 ± 1°C) restricted-access lab at the University of Auckland. Birds were weighed, banded with individual colour bands for easy identification and bled for genetic sexing (electronic supplementary material). The room was fitted with Sylvania 58 W Gro-lux lamps in a 12 h : 12 h light : dark cycle (lights-off at 18.00), four ceiling-mounted security cameras (Wisenet 20M IR Flat VDome 5MP 2.8 mm lens) and a Bioacoustics Audio Recorder (Frontiers Lab; sampling rate: 44.1 kHz, bit 24) to record the birds’ activity and vocalizations.

The birds were kept in identical individual cages (103 × 45 × 60 cm) in the lab, within auditory and visual contact of each other. Each cage had two perches, *ad libitum* water, and birds were fed dog food pellets once a day and fruit or mealworms (*Tenebrio molitor*) twice a week. Birds were held for five months before these experiments were conducted owing to COVID lockdown delays, which allowed habituation to laboratory conditions.

### (b) Experimental design

To understand the effects of SD on mynah vocal performance, we disturbed common mynahs' sleep during three different experiments: (i) full night SD (12SD; 18.00 to 06.00), (ii) first half of the night SD (6SDF; 18.00 to 00.00), and (iii) last half of the night SD (6SDL; 00.00 to 06.00). The experiments were performed between March and April 2021, with two weeks between each experiment to allow a full recovery.

Each disturbance experiment followed the same pattern. On night 1 (baseline), birds had an undisturbed night of sleep; on night 2 (SD), the birds were kept awake for either the whole night (12SD), first half of the night (6SDF) or the last half of the night (6SDL). Birds were kept awake using the gentle handling method: the lights were off, but two people remained in the room, moving around for the entire period [38,39]. For the people in the room to see whether the birds were falling asleep during the experiment, a small and dim night light was placed in a corner of the room, under a thin cloth and away from the birds. For most of the night, human presence in the room was sufficient to keep the birds awake; however, if birds tried to sleep, the gentlest intervention possible was used, escalating as needed from moving around more frequently to approaching the cage, to gently tapping the cage and making a quiet noise, and, at the most extreme, to gently touching the bird whenever they showed any rest behaviour (closing eyelids, tucking beak under wing/feathers, motionless for more than 30 s). This method has been shown to be adequate for full physiological sleep deprivation in Australian magpies and pigeons [18,38,39]. However, birds can enter a sleep state in just a few seconds from the cessation of SDs [40]. Therefore, without neurophysiological information, through tools such as electroencephalography (EEG) and electromyography (EMG), we were unable to confirm whether the common mynahs were fully sleep-deprived [41,42].

### (c) Sound data processing

The day after each treatment night (baseline, SD), we quantified the birds’ vocalizations using the video cameras and audio recorders placed in the room. Common mynahs have a variety of complex vocalizations which are used in different contexts [43]. When in groups, common mynahs often sing together, with individuals joining mid-chorus [44]. Since common mynahs are social birds, isolating them would likely cause stress and changes in behaviour [45]. Therefore, all bird vocalizations were recorded together.

Bird vocalizations are often classified into two categories: songs and calls. Songs are usually described as melodious, complex bouts of syllables, while calls are defined as simple, short elements [46,47], though the threshold can be subjective. Songs and calls are also expected to have different functions. Songs are primarily used for long-range communication, including mate attraction and territory defence [1,48]. Calls are short-range signals used for social communication, such as signalling danger or food (e.g. alarm calls) and maintaining social cohesion (e.g. contact calls) [47,49]. We focused on two types of common mynah vocalizations: contact calls and songs. We defined contact calls as short vocalizations (<1 s) with low amplitude, narrow bandwidth and frequencies from 1 to 3.5 kHz (electronic supplementary material, figure S1). All other vocalizations were considered a song, including all instances where the bird visibly vocalized (posture or open beak). Attributing calls to individuals was not possible since common mynahs do not adopt a visually detectable vocalizing behaviour when contact-calling (JGM, personal observation, 2020); thus, the call analyses do not contain individual bird information (bird ID). We assigned bird ID to song selections by comparing selected songs to video recordings and noting which birds showed singing behaviour (e.g. rhythmically opening beak, head-bobbing and throat-moving) during the sampled time.

To standardize all recordings, we normalized their peak amplitude to −3.0 dB and reduced the noise (noise reduction: 10 dB; sensitivity: 6.00; frequency smoothing: 3 bands) using Audacity version 3.1.3. We used Raven Pro 1.6.3 [50] to visualize spectrograms and manually select songs and calls.

### (d) Song and call output

We sampled vocalizations during the first 5 min of each half-hour (or each hour for songs during 6SDF), from when the lights came on at 06.00 until 12.35 h for songs and until 18.00 for calls. We investigated the effects of SD (12SD, 6SDF and 6SDL) on song output by summing the duration of the songs for individual birds per 5 min interval per treatment (including zeros). For call output, we summed the duration of calls from all birds (since ID could not be assigned) per 5 min interval per treatment (including zeros).

### (e) Call spectral parameters

Only high-quality calls (high signal-to-noise ratio and no overlapping vocalizations) were kept for spectral analysis (baseline 12SD (*n* = 927), SD 12SD (*n* = 693); baseline 6SDL (*n* = 534), SD 6SDL (*n* = 698); baseline 6SDF (*n* = 489), SD 6SDF (*n* = 1283)). Using the *warbleR* package v. 1.1.27 [51], we imported selection tables from all experiments (12SD, 6SDF and 6SDL) with the *imp_raven* function and made them into an extended selection table with the *selection_table* function. Minimum and maximum frequency were extracted using *freq_range*. Next, we used the *spectro_analysis* function (window length = 700, overlap = 95) to extract the acoustic parameters of each call, obtaining 26 different acoustic parameters.

To reduce collinearity, we removed highly correlated variables (>60%), preferentially retaining the more comprehensive spectral components (e.g. mean frequency over first quartile frequency); nine variables remained: duration, mean frequency, kurtosis, time entropy, entropy, dominant frequency range, modulation index, dominant frequency at the start of the signal, dominant frequency slope [51]. To further reduce the dimensionality of the dataset, we combined the remaining variables by performing principal component analysis (PCA). Two principal components (PCs) explained 46.1% of the variation among spectral parameters (electronic supplementary material, table S1). Dominant frequency range, entropy, duration and mean frequency were positively loaded onto PC1 (28.7%). PC2 (17.4%) was a combination of positive loading from the starting dominant frequency and negative loading from the dominant frequency slope.

### (f) Song complexity

For song-complexity analysis, we restricted our data to high-quality songs (high signal-to-noise ratio and only one bird singing) from the 12SD treatment that we could attribute to individual birds (baseline 12SD, *n* = 243; SD 12SD, *n* = 187). These songs were uploaded into the Web-based software Koe [52], and we audio-visually classified each syllable into six types: low grunt, buzz, repeated grunt, sweep, tone and wave. We assigned each syllable type a difficulty score from 1 to 6 (1 = least difficult, 6 = most difficult; electronic supplementary material, figure S2). Tonal syllables with more frequency modulations and repetitions were scored as more difficult than syllables with smaller bandwidth and low tonality [4]. We chose four measures of song complexity based on commonly used parameters [9,53–55]: syllable rate (syllables per second), syllable types (the number of different syllables in a song divided by the total number of syllables), switching (number of syllable type changes in a song) and average difficulty (summed syllable difficulty divided by the number of syllables in a song). To analyse song complexity, we calculated the average of each complexity measurement per bird per time sample (note that not all birds had a high-quality song per time sample).

### (g) Daytime activity observations

To determine the effect of SD on activity levels, we observed the birds’ daytime behaviour after baseline and SD days (12SD, 6SDF and 6SDL). We recorded the birds’ activities during the same periods as we recorded their songs—the first 5 min of each half-hour from when the lights came on, at 06.00, until 12.35. We summed all the time (in seconds) birds spent active or inactive during the 5 min period. Birds were considered ‘active’ when performing behaviours such as foraging, drinking, walking, jumping, flying, alertness, bowing, preening, calling and singing. Without neurophysiological measures of sleep (e.g. EEG and EMG), we were unable to determine sleep duration during the day; therefore, we focused on behavioural proxies of rest [42]. Postures indicative of rest behaviour included standing on one or both legs on a perch without movement, sitting with one or both eyes closed, tucking the head into feathers, or remaining motionless at the bottom of the cage.

### (h) Statistical analysis

We conducted all statistical analyses using the R programming software v. 4.2.1 [56]. Models were performed using the *lme4* package [57]. All model residuals were checked for normality, applying statistical transformations to the dependent variables when necessary. We performed *post hoc* tests using the function *emmeans* when fixed effects had a significant effect [58,59].

To test the effects of SDs on song output, we averaged the output of all three baselines for each individual in each time sample and compared them with the song output of each bird at each time sample after treatment nights. We performed a linear mixed model with song output (log-transformed) as the dependent variable, treatment (baseline, 12SD, 6SDF, 6SDL) as a fixed effect, and a random intercept and slope for experiment nested within individual birds. To test the effects of SDs on call output, we averaged the output of all three baselines for each time sample and compared them with the call output for each time sample after treatment nights. We performed a linear model with call output (log-transformed) as a dependent variable, and treatment (baseline, 12SD, 6SDF, 6SDL) as a fixed effect.

We investigated the effects of SD on call spectral parameters by performing linear models on the PC scores obtained through PCA analysis. The baselines from all three experiments were pooled and compared with the three treatments (12SD, 6SDF and 6SDL). The linear models included PC1 or PC2 as dependent variables and treatment as a fixed effect.

We explored the effects of SD on individual song complexity using linear mixed-effect models, with the mean complexity measurement (syllable rate, syllable diversity, switching and average difficulty) as dependent variable, the interaction between treatment and sex as fixed effects, and a random intercept and slope for experiment nested within individual birds.

To analyse the birds’ activity patterns after SD, we performed a linear mixed model with time active as the dependent variable (in seconds), treatment as a fixed effect, and a random intercept and slope for experiment nested within individual birds.

## 3. Results

### (a) Song output decreases after sleep disturbance

**Figure 1.**
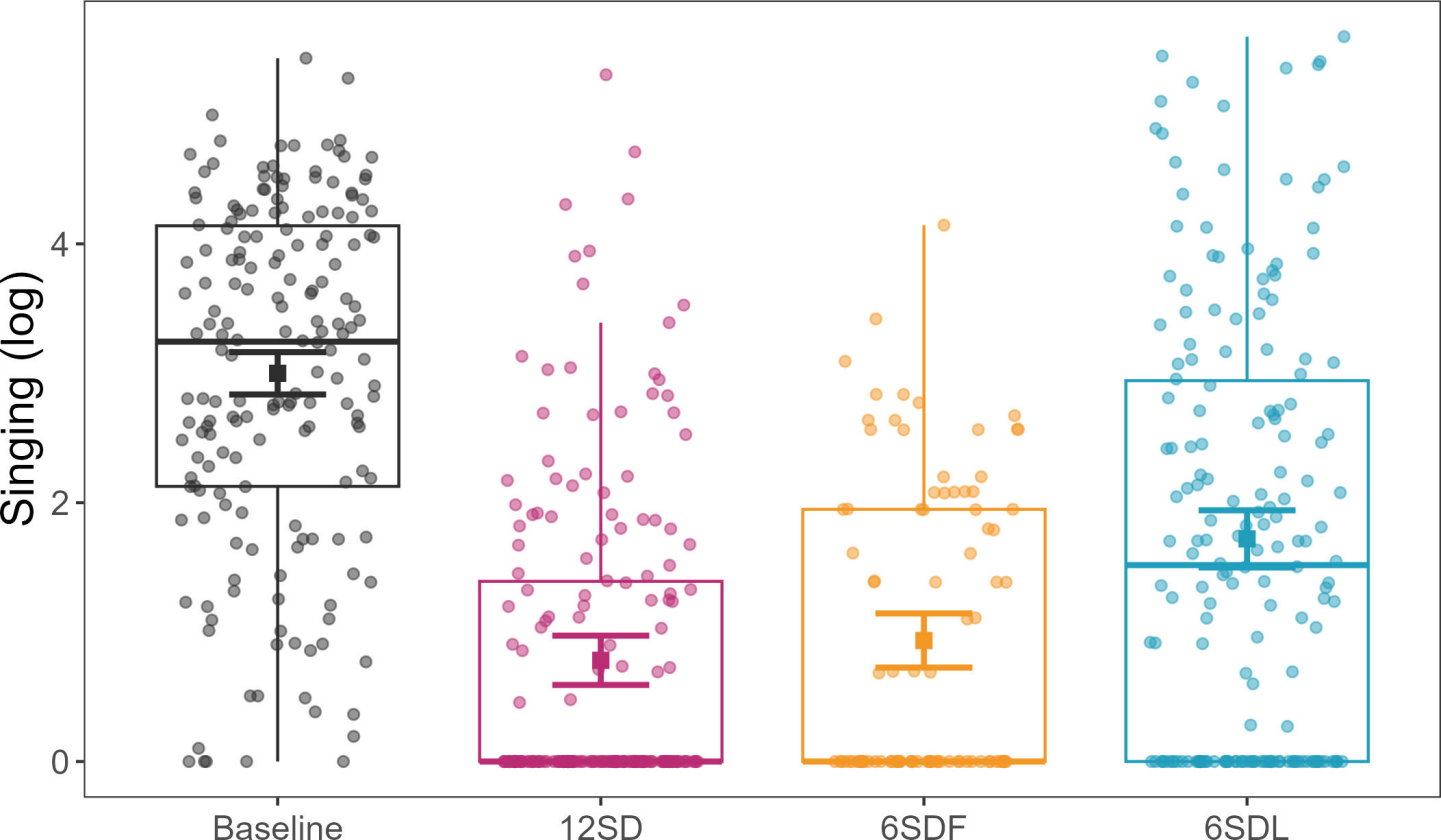
Effects of sleep disturbance (SD) treatments on song output (log-transformed). Treatments: baseline = black; first 6 h SD = yellow; last 6 h SD = blue; 12 h SD = pink. Points (jittered) represent the time spent singing (seconds logged) per individual bird (*n* = 13) per time sample. Squares represent the model’s predicted effects, with 95% confidence intervals.

Common mynahs sang less overall after all three disturbance treatments than after baseline nights (figure 1). After 12 h of SD, birds decreased singing the most (12SD: *β* ± s.e = −2.2177 ± 0.166, d.f. = 12.042, *t* = −13.357, *p* < 0.001), followed by the first 6 h SD (6SDF: *β* ± s.e = −2.065 ± 0.173, d.f. = 34.620, *t* = −11.955, *p* < 0.001) and the last 6 h SD (6SDL: *β* ± s.e = −1.279 ± 0.230, d.f. = 12.234, *t* = −5.573, *p* < 0.001), when compared with baseline. However, the effects of 12 h and the first 6 h SD were not significantly different from each other (*emmeans*: *β* ± s.e = −0.153 ± 0.165, d.f. = 12, *t* = −0.929, *p* = 0.790).

### (b) Call output does not change after sleep disturbance

**Figure 2.**
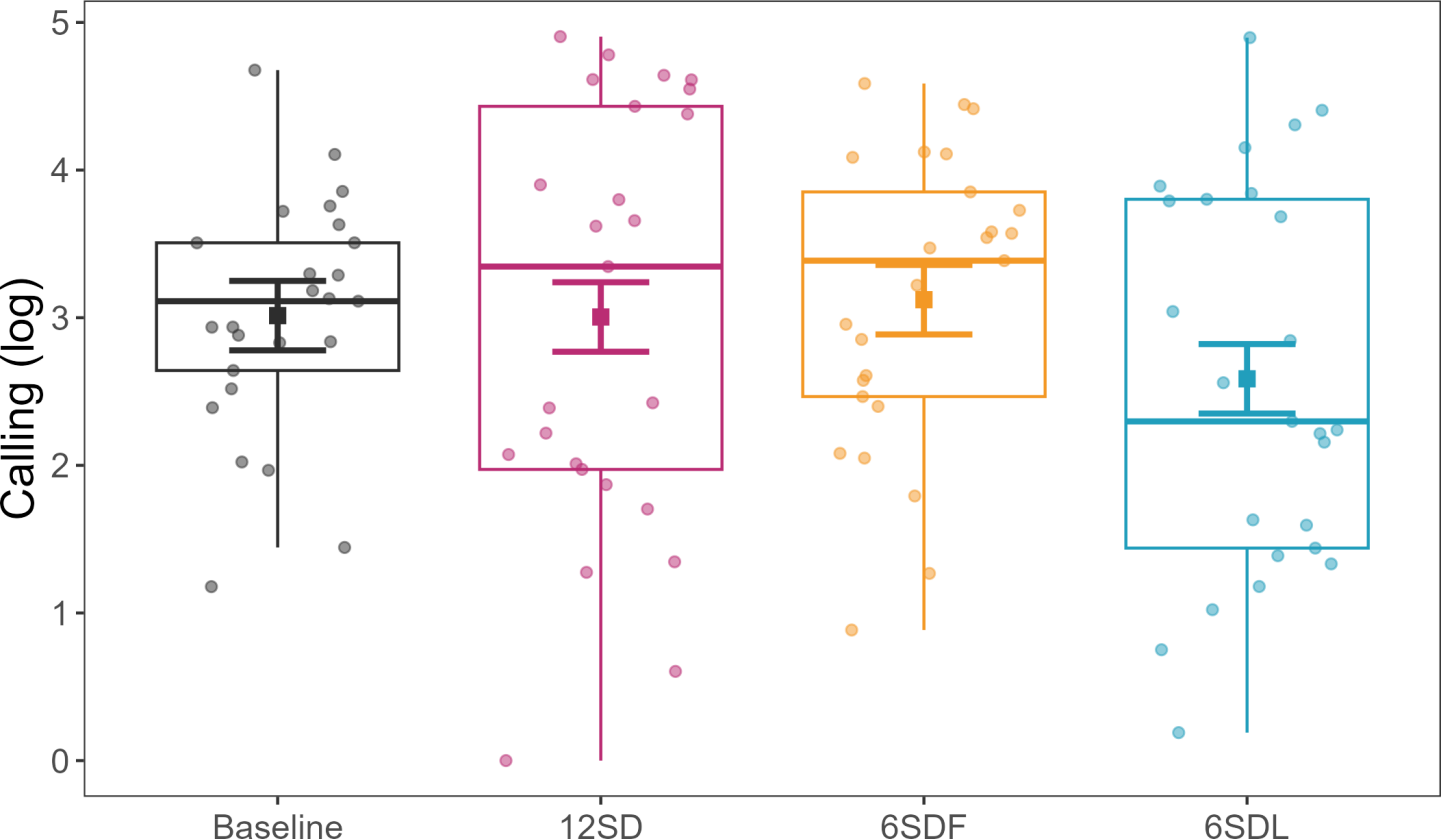
Effects of sleep disturbance (SD) treatments on call output (log-transformed). Treatments: baseline = black; first 6 h SD = yellow; last 6 h SD = blue; 12 h SD = pink. Points (jittered) represent the time spent calling (seconds logged) per time sample (all birds together). Squares represent the model’s predicted effects, with 95% confidence intervals.

When comparing the total amount of calling during treatments and baseline, mynahs called 63.88% more after 12SD, 25.65% more after 6SDF and 3.26% more after 6SDL than baseline. However, there were no statistical differences between call output during baseline and treatments when comparing samples (figure 2; 12SD: *β* ± SE= −0.009 ± 0.333, d.f. = 96, *t* = −0.028, *p* = 0.978; 6SDF: *β* ± s.e = 0.108 ± 0.333, d.f. = 96, *t* = 0.324, *p* = 0.747; 6SDL: *β* ± s.e = −0.428 ± 0.333, d.f. = 96, *t* = −1.286, *p* = 0.201).

### (c) Sleep disturbance affects call spectral parameters

SDs affected some of the measured spectral parameters of common mynah calls. PC1 scores after 12SD and 6SDF were higher than baseline (12SD: *β* ± s.e = 0.132 ± 0.069, d.f. = 4648, *t* = 1.897, *p* = 0.058; 6SDF: *β* ± s.e = 0.548 ± 0.056, d.f. = 4648, *t* = 9.715, *p* < 0.001), but lower after 6SDL (*β* ± s.e = −0.513 ± 0.068, d.f. = 4648, *t* = −7.516, *p* < 0.001). A high PC1 score indicates a higher dominant frequency range, mean frequency and entropy, and longer calls. PC2 scores were lower after all treatments (12SD, 6SDF and 6SDL) than baseline (12SD: *β* ± s.e = −0.207 ± 0.055, d.f. = 4648, *t* = −3.756, *p* < 0.001; 6SDF: *β* ± s.e = −0.296 ± 0.044, d.f. = 4648, *t* = −6.615, *p* < 0.001; 6SDL: *β* ± s.e = −0.198 ± 0.054, d.f. = 4648, *t* = −3.654, *p* < 0.001). A low PC2 score indicates a lower starting dominant frequency and a higher dominant frequency slope.

### (d) Song complexity

Common mynahs’ song complexity showed an interaction between treatment and sex after 12 h of SD in two of the four measured parameters (table 1). Female common mynahs sang at a lower syllable rate after SD than during baseline (*emmeans*: *β* ± s.e = −0.980 ± 0.331, d.f. = 20.30, *t* = −2.964, *p* = 0.035), while males showed no differences in syllable rate (*emmeans*: *β* ± s.e = −0.232 ± 0.206, d.f. = 6.02, *t* = −1.127, *p* = 0.688). Males showed a trend to sing fewer syllable types within a song after SD than after a baseline night (*emmeans*: *β* ± s.e = −0.132 ± 0.049, d.f. = 5.99, *t* = −2.691, *p* = 0.125), while females showed no differences in syllable types (*emmeans*: *β* ± s.e = 0.085 ± 0.079, = 19.59, *t* = 1.076, *p* = 0.708). There was no difference between the treatment and sex interaction on syllable switches or average difficulty (table 1). Additionally, for syllable rate, syllable types and average difficulty, there was a very low individual bird and experiment variance compared with residual variance.

**Table 1.** Summary of linear models results on the effect of 12 h sleep disturbance on the four measured song-complexity parameters. The reference level of all models is female baseline. *Post hoc* comparisons of the significant variables are described in the text. Bold numbers indicate *p* < 0.05*.*

	predictors	treatment	sex	treatment: sex	residual variance	bird variance	experiment variance
syllable rate	estimates (s.e.)	−0.97 (0.32)	−0.23 (0.26)	0.74 (0.37)	0.563	0.058	0.009
	CI	−1.60 −0.36	−0.75 0.28	0.02 1.48			
	d.f.	81.75	13.35	72.26			
	*s*tatistic	−3.08	−0.88	2.00			
	*p*	**0.003**	0.394	**0.049**			
syllable types	estimate (s.e.)	0.09 (0.08)	0.06 (0.06)	−0.22 (0.09)	0.033	0.003	0.000
	CI	−0.06 0.23	−0.06 0.17	−0.39 −0.04			
	d.f.	83.05	12.61	86.95			
	statistic	1.12	0.94	−2.43			
	*p*	0.266	0.365	**0.017**			
syllable switches	estimates (s.e.)	−0.344 (0.39)	0.07 (0.31)	−0.02 (0.47)	0.703	0.128	0.176
	CI	−1.10 0.42	−0.56 0.69	−0.90 0.94			
	d.f.	8.88	6.62	5.61			
	statistic	−0.89	0.21	0.04			
	*p*	0.398	0.843	0.971			
average difficulty	estimate (s.e.)	0.11 (0.26)	−0.03 (0.18)	−0.24 (0.30)	0.400	0.013	0.011
	CI	−0.39 0.63	−0.38 0.34	−0.85 0.36			
	d.f.	90.00	90.00	90.00			
	statistic	0.44	−0.16	−0.80			
	*p*	0.658	0.869	0.427			

### (e) Daytime resting activity

**Figure 3.**
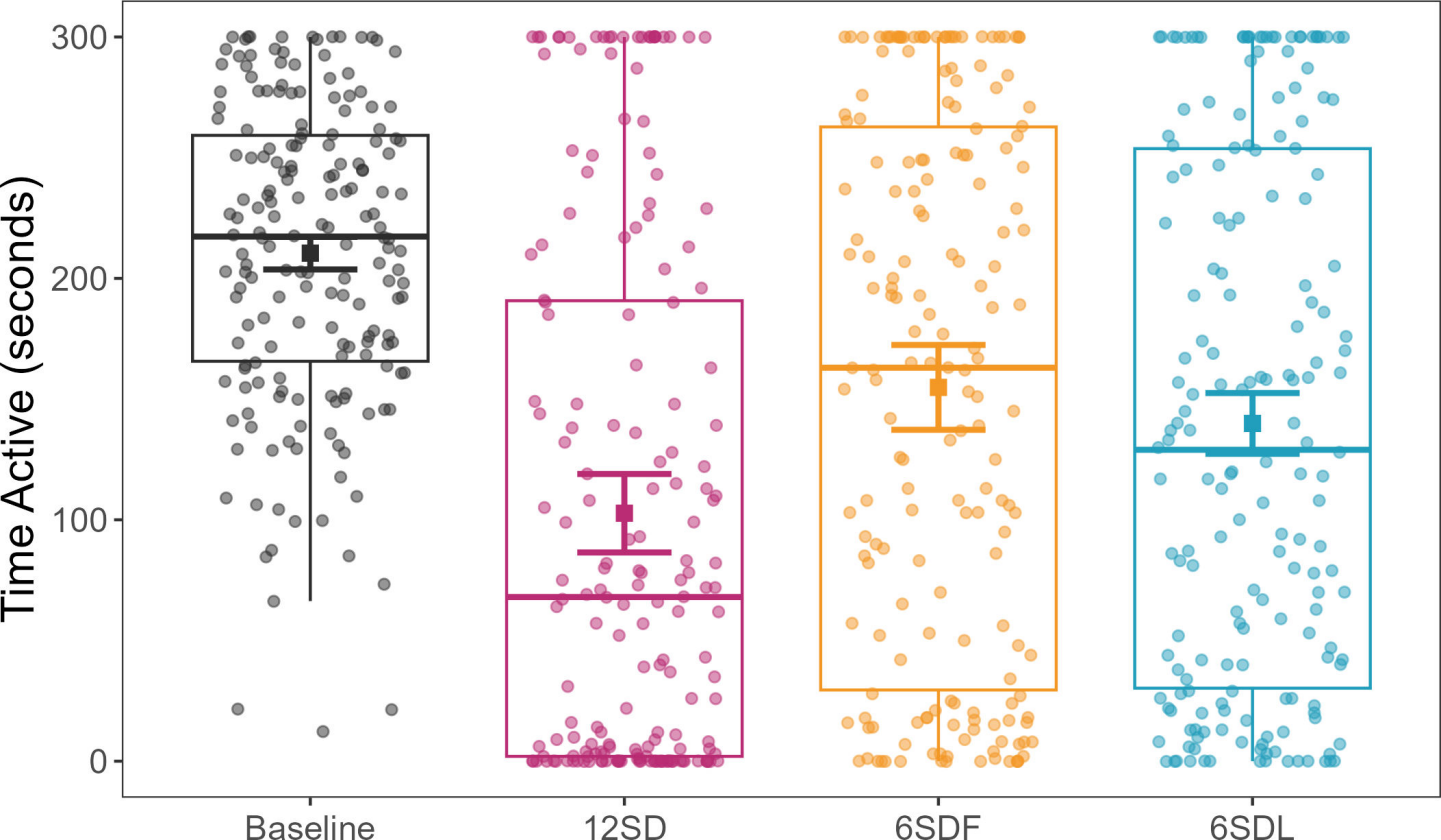
Effects of sleep disturbance (SD) treatments on bird activity. Treatments: baseline = grey; first 6 h SD = yellow; last 6 h SD = blue; 12 h SD = pink. Points (jittered) represent the time spent active per individual per time sample (total observation per sample = 300 s). Squares represent the model’s predicted effects, with 95% confidence intervals.

Common mynahs spent less time active (more time resting) overall after all three disturbance treatments than after a baseline night (figure 3). The largest decrease in activity was found after 12 h of SD (12SD: *β* ± s.e = −107.729 ± 17.602, d.f. = 16.397, *t* = −6.120, *p* < 0.001), followed by last 6 h SD (6SDL: *β* ± s.e = −70.531 ± 14.287, d.f. = 15.810, *t* = −4.937, *p* < 0.001) and first 6 h SD (6SDF: *β* ± s.e = −55.597 ± 18.824, d.f. = 15.778, *t* = −2.954, *p* = 0.009), when compared with baseline. However, the effects of the first 6 h and the last 6 h SD were not significantly different from each other (*emmeans*: *β* ± s.e = 14.9 ± 15.4, d.f. = 12, *t* = 0.968, *p* = 0.770).

## 4. Discussion

Our findings show that short-term SD affects common mynahs’ vocal performance across numerous metrics. Supporting our predictions, common mynahs sang fewer and less complex songs after SDs when compared with baseline. However, contrary to our predictions, they increased the total percentage of time spent calling (although not statistically significant), and these calls exhibited spectral differences after treatment nights. The effects of a full night and half a night of SD on vocal performance and activity were generally similar in type but different in magnitude; as predicted, effects after a full night of SD were larger than disturbance for half of the night. While, for song output, disturbance in the first half of the night had larger effects than the last half of the night, bird’s daily activity was more affected after the last half of the night when compared with the first half.

### (a) Song output and daytime activity

Previous studies in humans and one bird species (Australian magpies) have shown a reduction in communication output (the number of words/songs) after a night of sleep deprivation [18,27]. The extent of the effects also depends on the length of SD—the number of sleepless nights has an increasing effect on vocal performance [30]. Our results corroborate these findings: in common mynahs, SDs decreased singing output, and a full night of SD had a stronger impact on song than half a night. Additionally, common mynahs’ response to SD was different depending on what part of the night it occurred. Birds sang less when their sleep was disturbed in the first half of the night (6SDF) than in the second half (6SDL). Therefore, it is expected that increasing the number of days of sleeplessness would also increase the effect on song output. Nonetheless, the outcome of SD is unlikely to be linear. During more extended periods of sleeplessness, birds might adapt to the lack of sleep and compensate by changing the time of singing or by increasing individual song duration, for example. Sleep-deprived Australian magpies (deprived for 12 h) sang fewer but longer songs and shifted their singing time to later in the day so that the total amount of singing per day was not affected [18].

Lack of sleep has been shown to negatively affect motivation and effort allocation [18,60,61]; thus, individuals might prioritize recovering sleep over communication. In this study, common mynahs indeed spent more time resting during the day after SD, especially after a full night of disturbance. Additionally, after SD, birds were less likely to sing at all than they were at baseline. This corresponds to other studies that show that after deprivation, birds attempt to recover sleep by sleeping during the day, and that longer periods of sleep deprivation result in more daytime sleep [18,38,39]. Common mynahs rested more after having their sleep disturbed for the last half of the night, when compared with the first half. Since SD during the first half of the night allows birds to recover some sleep at nighttime, it is logical to infer that birds that are sleep-disturbed during the latter half would rest more during the daytime. However, the results with song output showed the opposite of the activity results—birds sang less after SDs in the first half of the night compared with the last half of the night. Additionally, we observed a trend of mynahs spending more time calling after SDs than after baseline. However, when accounting for when times samples were collected, the call results were not statistically significant. Our findings indicate that sleepiness and lack of motivation might not be the only mechanisms behind the effects of SD on vocal performance.

Sleep stages are usually associated with different times of the night, with non-rapid eye movement sleep (NREM) being more predominant in the first half of the night, while rapid eye movement sleep (REM) occurs mostly in the latter half of the night [39,62]. While the exact functions of each sleep stage are still unknown, there is evidence that REM and NREM sleep possess distinct and complementary functions [63–66]. Thus, lack of sleep might affect performance differently depending on when it occurs. Studies in humans have shown that sleep deprivation in the last half of the night (when REM occurs) negatively affects physical performance [67], while sleep deprivation in the first half of the night (when NREM is prevalent) negatively affects declarative memory performance [68,69]. However, this relationship is likely complex in birds. After disturbances in the first half of the night, Australian magpies increase NREM sleep and decrease REM in the second half of the night when compared with a normal night; in contrast, European starlings show no change in sleep stage percentages [19,39,70]. It is important to note that, in this study, we found some differences in song output between the baseline days. While the baselines from 12SD and 6SDF were not statistically different, birds sang more during baseline 6SDL when compared with the other two days (electronic supplementary material, figure S3). Although this is unlikely to explain the much larger differences observed between baseline and treatments, it could partially explain the less pronounced difference found in the song output after 6SDL when compared with 12SD and 6SDF. Therefore, more extensive research is needed to identify whether the differences in song output after disturbances in the first versus the last half of the night are due to sleep stages.

### (b) Call output and spectral parameters

We had predicted that common mynahs would be less motivated to call and would decrease calling output after SD. However, although mynahs were less active after SD, they increased the total amount of time spent calling (not statistically detectable when comparing time samples). Animals generally use contact calls for individual recognition and to maintain social cohesion, especially in areas with low visibility [47,49]. Since sleep-disturbed birds rested more during the day, their perception of vulnerability and danger may have increased. Therefore, contact calling may have helped restore group cohesion and indicated to the group that it is safe to sleep. However, while SD in the first half of the night also increased call output (not statistically detectable), disturbance in the latter half of the night did not affect call output.

Bird contact calls are usually simple vocalizations that are, in principle, innate and remain unchanged through adulthood [47]. Nonetheless, we found temporal and spectral differences in calls after 6 and 12 h of SD; common mynahs vocalized longer calls with a lower frequency and narrower range of frequencies. This is concerning, since contact calls are also used for recognition and often differ between individuals [49]. Long-tailed tits, for example, use frequency parameters to identify kin contact calls [71]. When frequency parameters from kin calls are manipulated, they respond as if they were made by a non-kin individual [72]. Additionally, calls with a narrow bandwidth and longer duration degrade less, being better transmitted over longer distances [73]. Therefore, the call frequency changes caused by SD could affect common mynahs’ social behaviour, group cohesion and, subsequently, their fitness.

In our study, we were unable to identify which birds were calling. Hence, the changes found could result from specific birds calling more/less often after SD, thus changing the group’s mean parameters. Additionally, without individual identification, we could not account for repeated measures in the call statistical models. However, there is increasing evidence of high plasticity of bird calls [47,74–76]. Therefore, future studies should also consider investigating spectral changes in calls due to SDs.

### (c) Song complexity

Humans often show reduced vocal complexity when sleep-deprived, speaking simpler sentences and repeating the same words more often [27,29]. To our knowledge, ours is the first study to analyse the relationship between SD and vocal complexity in non-human animals. Common mynahs sang less complex songs after 12 h of SD. It is important to recognize that this effect could be driven by the lower number of good-quality songs (high signal-to-noise ratio and only one bird singing) sung, thus reducing the power to detect complex songs. Additionally, the low variance attributed to individual birds suggests that song complexity does not appear to be a consistent feature of any individual. In other words, no bird seems to consistently produce higher- or lower-quality songs than others. This could reflect the number of songs sampled per individual, or it may indicate that birds have learned from one another, leading to a convergence in song quality across the group. However, reduced complexity is predicted based on previous research and our understanding of how memory and sleep interact. While animals sleep, new and old memories (which include songs) are consolidated and stabilized [77]. Evidence of memory consolidation in birds includes motor and neuronal replays of songs during sleep [78]. Birds activate neurons and syringeal muscles while asleep, following the same song patterns as when awake, including the type and order of specific syllables [79–81]. Therefore, SD in birds could lower their motivation to sing complex songs and disrupt song rehearsal at night, affecting song structure and complexity.

There is currently little information on the effects of a lack of sleep on vocal complexity. However, many studies have observed song performance changes in urban environments. Birds in areas with high levels of noise pollution have lower syllable rates, longer inter-syllable intervals and modified frequency parameters [75,82]. Noise and light pollution at night disrupts and lightens sleep in birds [19,20,83]. The changes in vocal complexity associated with increased light and noise at night might partially be an indirect effect of SDs. However, even without anthropogenic disturbances, birds’ sleep can also be affected by seasonal changes such as extreme temperatures, migration, breeding and parental duties [42]. The loss of song complexity is likely to have important fitness consequences. Because song complexity can be an honest signal of nutritional condition, health and cognition [84–86], birds with more complex songs are often better at protecting territory, attracting mates and maintaining dominant hierarchical positions [15,87–90]. Sleep-deprived birds singing lower-quality songs could be impacted on resource and mate acquisition.

## 5. Conclusions

These findings reveal that short-term SD can affect how much and how individual birds vocalize. The objective of this study was to test general predictions regarding the effects of SD on adult bird vocal performance [17]. Twelve hours of SD appears to have stronger effects on vocal performance parameters than 6 h, and SD during the first 6 h influences vocal performance more than disturbance during the last 6 h. This is consistent with human studies, which show that more extended periods of SD have stronger effects on diurnal behaviour [30]. While the mechanisms behind these changes are yet unknown, it is unlikely to be solely due to stress caused by SD methods. A recent study on common mynahs with elevated corticosterone showed the opposite result to the reported finding here—when corticosterone was elevated, birds increased singing and song complexity and decreased call rates [55].

This is the first study that directly investigates changes in song complexity due to a lack of sleep. Thus, we do not have any *a priori* research on which to base a decision regarding which parameters are most likely to be affected. Since song-complexity parameters are seldom concordant [53], we performed an exploratory analysis and tested commonly used song-complexity parameters separately. However, with numerous tests, the probability of a type I error (false positive) increases. Given that, caution is warranted in interpreting the complexity results, and future studies should seek to test specific parameters based on our findings.

Results such as these are essential to understanding the impacts of SDs at night, such as light and noise pollution, and potentially inform policymakers to mitigate these effects. Therefore, more research with wild populations and different levels of ecologically relevant SDs will shed light on the importance of sleep for vocal performance in birds and will help to understand the consequences of a lack of sleep on vocalizations and their underlying mechanisms.

## Data Availability

The data that support the findings of this study are openly available in Figshare at [59]. Supplementary material is available online [91].
